# Selective anti-cancer effects of cannabidiol and Δ9-tetrahydrocannabinol via PI3K/AKT/mTOR inhibition and PTEN restoration in ovarian cancer cells

**DOI:** 10.3389/fphar.2025.1693129

**Published:** 2025-12-15

**Authors:** Siyao Tong, Watcharin Loilome, Nisana Namwat, Poramate Klanrit, Arporn Wangwiwatsin, Zar Zar Win, Preeya Koyabuth, Bandit Chumworathayi

**Affiliations:** 1 Department of Systems Biosciences and Computational Medicine, Faculty of Medicine, Khon Kaen University, Khon Kaen, Thailand; 2 The First Affiliated Hospital of Jinzhou Medical University, Jinzhou, China; 3 Cholangiocarcinoma Research Institute, Khon Kaen University, Khon Kaen, Thailand; 4 Department of Obstetrics and Gynaecology, Faculty of Medicine, Khon Kaen University, Khon Kaen, Thailand

**Keywords:** ovarian cancer, cannabidiol (CBD), tetrahydrocannabinol (THC), PI3K/AKT/mTOR, PTEN

## Abstract

**Introduction:**

Ovarian cancer is a highly lethal gynecological malignancy, often diagnosed at advanced stages. Cannabidiol (CBD) and delta-9-tetrahydrocannabinol (THC) demonstrate anti-tumor activity in various cancers including ovarian cancer through multiple signaling pathways and are increasingly explored as adjuncts to chemotherapy. However, the effects of CBD and THC combination treatment and its specific mechanisms remain unclear. This study evaluated the anti-tumor effects of CBD, THC, and their combination on SKOV3 and A2780 ovarian cancer cells, focusing on phosphorylation-dependent regulation of the PI3K/AKT/mTOR pathway.

**Methods:**

SKOV3, A2780, and IOSE cells were treated with CBD, THC, and equimolar CBD: THC combinations. Cytotoxicity was assessed using Sulforhodamine B assay, while synergistic interactions were analyzed by the Chou-Talay method using CompuSyn. Cell cycle distribution and apoptosis were evaluated, and phosphorylation of PI3K, AKT, mTOR, and PTEN was examined by Western blotting.

**Results:**

The CBD: THC combination treatment showed potent, selective cytotoxicity at 48 h, with lower IC_50_ values than in non-tumor IOSE80 cells. The Chou–Talalay method validated a synergistic effect between CBD and THC. The combination treatment induced cell cycle arrest and enhanced apoptosis. Western blot analysis exhibited that equimolar CBD: THC (2.5:2.5 μM) markedly reduced phosphorylation of PI3K, AKT, and mTOR, while increasing phosphorylation of PTEN, thereby reactivating tumor-suppressive signaling.

**Conclusion:**

These findings highlight that CBD: THC combination treatment effectively inhibited ovarian cancer cell growth and invasion via oncogenic PI3K/AKT/mTOR signaling and reactivates PTEN. The combination may represent a promising targeted therapeutic approach, warranting further *in vivo* validation to elucidate its clinical potential.

## 1 Introduction

Gynecological malignancies, comprising cancers of the cervix, ovaries, vagina, vulva, and uterus, represent a significant health concern within the female population (Zhu et al., 2024, p. 202). Of these, ovarian cancer is the most critical burden, presenting the highest rates of both morbidity and mortality worldwide due to its silent nature and non-specific symptoms in early stage diagnosis (Siegel et al., 2018). The global figures suggest that over 295,000 patients were diagnosed with ovarian cancer, and approximately 185,000 women died from it (Bray et al., 2018), underscoring the unmet medical need and ongoing unfavorable outcomes in the fight against gynecological cancers. Despite advancements in standard strategies such as surgery intervention and applying platinum-based compounds such as cisplatin and carboplatin to combat cancer by cross-linking DNA and inducing apoptosis (Marth et al., 2017; Zoń and Bednarek, 2023), the common development of drug resistance, considerable side effects, and tumor recurrence remains a major challenge, indicating the fact that the exploration of alternative or adjunctive therapeutic regimes with potential anticancer properties and improved biocompatibility becomes the major clinical problems that need to be addressed (Hanker et al., 2012; Hsu et al., 2015; Mihanfar et al., 2019).

Research indicates that the antiproliferative potential of natural products against ovarian cancer cells has been documented, with various actions including apoptosis induction, cell cycle arrest, and angiogenesis inhibition (Pistollato et al., 2017). This herbal-based therapy strategy supports the potential use of natural products to improve conventional therapies and develop more effective treatments for ovarian cancer. Among herbal-based compounds, cannabinoids are diverse bioactive constituents, commonly found in the Cannabis sativa plant. Recently, cannabinoids, especially a non-psychoactive compound called cannabidiol (CBD), and the principal psychoactive constituent of cannabis known as delta-9-tetrahydrocannabinol (THC) have gained popularity, exhibiting a favorable pharmacological profile (Chadwick et al., 2020), including antioxidant, anti-inflammatory, and notably, anti-tumor properties (Ligresti et al., 2006; Rajesh et al., 2010; Takeda et al., 2013; López-Valero et al., 2018; Jastrząb et al., 2019; Li et al., 2022; Erzurumlu et al., 2023; Zaiachuk et al., 2023).

Reports have been documented that CBD may play an important role to modulate cancer cells through various mechanisms including inhibition of cell proliferation, induction of ROS-mediated apoptosis, suppression of angiogenesis in various cancer types including prostate, skin, lung, glioblastoma and breast cancer cells (Ligresti et al., 2006; Ramer et al., 2012; Velasco et al., 2012; Baram et al., 2019; Hinz and Ramer, 2022). Furthermore, THC, despite being known for its psychoactive properties, has also exhibited potential anticancer activity, with cell type-dependent and dose-dependent manners (Braun et al., 2024; Younes et al., 2024). Notably, few research showed that the combinatory application of THC and CBD may offer a synergistic or additive therapeutic benefit while potentially mitigating the psychoactive effects of THC alone (Kovalchuk and Kovalchuk, 2020; Younes et al., 2024). However, most of these studies have been conducted in non-gynecological cancer models, and evidence regarding the efficacy or mechanisms of this CBD: THC combination in ovarian or other gynecological cancers remain scarce. Therefore, the precise molecular mechanisms by which these cannabinoids and their combinations exert their effect on ovarian cancer cell lines remain to be elucidated.

Despite growing evidence that cannabinoids such as CBD and THC can modulate cancer cell proliferation and survival, the molecular basis of these effects in ovarian cancer, particularly in relation to the PI3K/AKT/mTOR pathway and its upstream regulator PTEN, remain largely unexplained. The PI3K/AKT/mTOR signaling axis is frequently overactivated in ovarian cancer and plays a key role in promoting cell proliferation, survival and chemoresistance, with PTEN functioning as a key negative regulator of this pathway. However, the effects of CBD, THC, and their combination on the regulation of this signaling cascade and PTEN activity in ovarian cancer cells remain unclear. This knowledge gap highlights the need for mechanistic studies to elucidate how cannabinoids restore tumor suppressor signaling and counteract oncogenic PI3K/AKT/mTOR activation.

Our study aims to bridge a knowledge gap by elucidating the individual and combined effects of CBD and THC on the proliferation, clonogenicity, cell cycle progression, and apoptosis of human ovarian cancer cell lines, while providing mechanistic insight into their influence on the PI3K/AKT/mTOR pathway and PTEN regulation. We conducted a platinum-sensitive model, A2780 cells and an innate platinum resistance SKOV3 cell line, thus providing a relevant *in vitro* model to evaluate the potential of cannabinoids in different ovarian cancer types. In addition, we included non-tumor IOSE cells to assess the selective cytotoxicity for the compounds. The findings from this study could significantly advance the understanding of cannabinoid-based mechanisms in ovarian cancer, potentially facilitate future *in vivo* investigations and ultimately, the development of novel therapeutic interventions aimed at patient outcomes.

## 2 Materials and methods

### 2.1 Cell lines and cell culture

The human ovarian cancer lines including SKOV3 (BNCC-310551), A2780 (BNCC-351906) and non-tumor IOSE cell lines were purchased from Bena Cell Culture (BNCC, Beijing, P.R.China). The cell lines were cultured in RPMI nutrient mixture supplemented with 10% FBS and 100 IU/mL of penicillin-streptomycin at 37 °C containing 5% CO_2_ in a humidified incubator. The presence of *Mycoplasma* contamination was periodically checked.

### 2.2 Chemicals, antibodies, and reagents

The cannabidiol (CBD) and delta-9-tetrahydrocannabinol (THC) are kindly provided by Prof. Bandit Chumworathayi from the Department of Obstetrics and Gynaecology, Khon Kaen University, Thailand. Both CBD and THC at a concentration of 5 µM were used, based on preliminary IC_50_ measurements showing similar cytotoxicity against ovarian cancer cells. This concentration allows for direct comparison of individual and combined effects and falls within the biologically relevant, non-toxic range. Although CBD has a lower affinity for CB1 and CB2 receptors than THC, its anticancer effects also involve receptor-independent mechanisms, thus supporting the use of equimolar doses in combination studies. Moreover, this approach has been used in previous studies investigating the comparative or combined effects of CBD and THC in cell-based systems (Zhornitsky and Potvin, 2012; Scott et al., 2017). Sulforhodamine B (SRB), Annexin V-FITC/PI Apoptosis Detection Kit, Cell Cycle and Apoptosis Analysis Kit (PI staining), JC-1 and MitoSOX Red were purchased from MedChemExpress (MCE) (Monmouth Junction, NJ, United States). Roswell Park Memorial Institute (RPMI) 1,640 medium were purchased from BNCC, China. Mogengel Matrix were purchased from Xiamen Mogengel Biotechnology Co., Ltd. (Xiamen, China). Antibodies used for Western blotting were as follows: rabbit anti-mTOR (Cell Signaling Technology, Danvers, MA, United States, Cat# 2983), rabbit anti-Akt (Cell Signaling Technology, Cat# 4691), rabbit anti-PTEN (Cell Signaling Technology, Cat# 9559), rabbit anti-phospho-Akt (Cell Signaling Technology, Cat# 4060), and rabbit anti-GAPDH (Cell Signaling Technology, Cat# 5174), rabbit anti-PIK3CA (Thermo Fisher Scientific, Waltham, MA, United States, Cat# PA585091), rabbit anti-phospho-PI3K p85 alpha (Tyr607) (Thermo Fisher Scientific, Cat# PA5104853), rabbit anti-phospho-mTOR (Ser2448) (Thermo Fisher Scientific, Cat# MA533138), and rabbit anti-phospho-PTEN (S380/T382/T383) (ABclonal Technology, Wuhan, China, Cat# AP1346).

### 2.3 Cytotoxicity assay

Two thousand cells were seeded into 96-well culture plates (Corning, United States) for 12–16 h. After incubation, the medium was removed and replaced with different concentrations of CBD, THC, and combination in culture medium. After 24, 48, and 72 h of treatment, each plate was collected to process for SRB assay. Then, the cell viability was determined by SRB assay (Vichai and Kirtikara, 2006). Briefly, cells were fixed by layering 100 µL of ice-cold 10% trichloroacetic acid (TCA, Aldrich Chemical) on top of the growth medium. Cells were incubated at 4 °C for 1 h, after which plates were washed three times with distilled water, excess water drained off and the plates left to dry in air. SRB stain (50 μL; 0.4% in 1% acetic acid) was added to each well for 1 h. Subsequently, to remove excess dye, the wells were rinsed three times with 1% acetic acid. The plates were dried and 100 µL of 10 mM Tris base [tris (hydroxy methyl) aminomethane, pH 10.5] was added to each well to solubilize the dye. The plates were shaken gently for 1 h on a shaker (ZHW-334, SUMER LAB, Wuchang, China). The absorbance (OD) of each well was read at 510 nm using the Tristar 5 microplate reader and each assay was performed in triplicate. The combination index was calculated using the Chou–Talalay method with CompuSyn software (ComboSyn, Inc., United States).

### 2.4 Colony formation assay

To analyze the sensitivity of cells to CBD and THC treatment, we used a colony formation assay adapted from previously published articles (Franken et al., 2006; O’Reilly et al., 2023) with some modifications. Briefly, 500 cells were seeded at a density of 500 cells per well in a 6-well plate (2 mL/well) and incubated for 72 h. Prior to drug treatment, cells were allowed to adhere and establish small colonies for 72 h to ensure uniform cell attachment and initial proliferation. This modification to the standard protocol was intended to reduce variability and better reflect the effects of drugs on established colonies rather than single cells. Then, the old medium was removed and replaced with fresh medium containing different concentrations of CBD, THC, and CBD: THC combinations and cultured for 7 days. The cells were washed twice with PBS, and then the colonies were fixed with 4% paraformaldehyde for 20 min. The cells were stained with 0.5% crystal violet for 20 min at room temperature. Then, the cells were washed three times with PBS, dried upside down, and colonies of 50 or more cells were counted and analyzed using ImageJ software v1.54.

### 2.5 Transwell migration and invasion assay

Cell migration and invasion were assessed using Boyden chambers consisting of Transwell membrane filter inserts (Cat # 3422, Corning Costar). For migration, in brief, 200 μL of serum-free RPMI media with 5 × 10^4^ cells were seeded into upper chamber of each 24-well Transwell chamber (8 μm pore size), while the lower chamber with 10% FBS containing RPMI media. Next, the control, CBD, THC, and CBD: THC were added to the upper chamber of Transwell plates (8 μm; Corning, Tewkesbury, MA, United States). The Transwell chambers were incubated in a 37 °C incubator to allow cells to migrate to the lower surface of the filter. Migration and invasion cultural period were 48 h and cells that did not penetrate the filter were wiped off, and cells on the lower surface of the filter were fixed with 4% paraformaldehyde for 20 min and stained with 0.1% crystal violet. The numbers of invading cells were counted at 48 h under a light microscope from five random fields in a single chamber and invasive cells were measured at 48 h and quantified using ImageJ software v1.54g. For invasion assay, Matrigel was used as a pretreatment on Transwell chambers, then dried for 3 h at 37 °C. Similar steps were performed as with the Transwell migration experiment.

### 2.6 Flow cytometry analysis

Ovarian cancer cell lines (A2780 and SKOV3) and non-tumor IOSE cell lines were seeded at 2.5 × 10^5^ cells in a 10 cm dish and cultured overnight after which the cells were treated with 5 µM of CBD or THC and combination ratio of 2.5:2.5 µM of CBD: THC or control medium for 48 h. All cells were then resuspended in pre-chilled 1× binding buffer, stained with FITC and PI for 15 min at room temperature in the dark. The cell cycle distribution was then detected by staining DNA with propidium iodide, while apoptosis and necrosis were detected using an Annexin V-FITC/PI Apoptosis Kit. The cell cycle distribution and apoptosis were determined by flow cytometry (BD FACSCanto™ II, BD Biosciences, CA, United States). Cell cycle analysis was performed using ModFit LT software (Version 5.0; Verity Software House), while apoptosis data were analyzed with FlowJo™ v10.9 software (BD Life Sciences).

### 2.7 Determination of mitochondrial membrane potential (MMP ΔΨm)

To evaluate the effect of cannabinoids on the mitochondrial membrane potential, Tetraethylbenzimidazolylcarbocyanineiodide (JC-1) dye staining was carried out. Cells were seeded in a 24 well plate and after treatment with cannabinoids was given for 48 h to the cells. Then after PBS washing, cells were stained using JC-1 dye for 30 min in dark at 37 °C (Abalos et al., 2021). Finally, cells were washed twice with PBS, the dye was removed and assessed by flow cytometry. Fluorescence in cells was measured FlowJo™. The disturbance in MMP was observed based on the reduction in j-aggregates to j-monomers.

### 2.8 Determination of cellular ROS

The intracellular levels of mitochondrial reactive oxygen species (ROS) were analyzed using MitoSOX Red according to the manufacturer’s instructions. After treatment with cannabinoids, cells were incubated with 5 µM MitoSOX for 15 min at 37 °C in the dark. After washing 3 times with warm DMEM, we detected the ROS intensity using flow cytometry and analyzed by FlowJo™.

### 2.9 Western blotting

After treatment with CBD, THC, and combination, the cells were then lysed in NP-40 lysis buffer (150 mM NaCl, 50 mM Tris pH 8.0% and 1% NP-40) at 4 °C for 10 min and 12,000 rpm centrifuge at 4 °C for 10 min. After collecting the supernatants, the BCA assay (Thermo Fisher Scientific, IL, United States) was used to determine the protein concentrations. Samples containing equal amounts of protein (20 µg) were boiled for 5 min and separated on 10% SDS-PAGE gels. Samples were transferred onto polyvinylidene fluoride membranes (Millipore, Billerica, MA). After blocking with 5% bovine serum albumin (BSA) in Tris-buffered saline (TBS) at room temperature for 1 h, the primary antibodies (mTOR, PTEN, Akt, PI3K, phospho-mTOR, phospho-PTEN, phospho-Akt, phospho-PI3K) were incubated overnight at 4 °C.

After washing with TBS containing 0.1% polyoxymethylene sorbitan monolaurate (Tween-20 or TBS-T), the membranes were further incubated with horseradish peroxidase-conjugated secondary antibody (Invitrogen, CA, United States) at room temperature for 1 h. GAPDH was used as a loading control. After washing with TBS-T, the membranes were induced to the enhanced ECL Prime Western blotting Detection System (GE Healthcare, United Kingdom). The probed proteins on membranes were visualized by the chemiluminescence method using the ImageQuantTM analysis system (GE Healthcare, United Kingdom) in the dark. The densities of the bands were determined using ImageJ software. Experiments were performed at least 3 times from cell treatment.

### 2.10 Statistical analysis

GraphPad 10.4.0 software was used for statistical analysis, and all data are presented as the mean ± SD from at least three independent experiments. One-way ANOVA was used for experiments involving cell viability, clonogenic analysis, apoptosis, MMP, and ROS, with Tukey’s or Dunnett’s post-hoc test performed depending on the experimental design. Two-way ANOVA was used for experiments involving two variables, such as different treatment conditions and cell cycle stages (cell cycle analysis), cell lines and treatment groups (Western blot), or combined treatment groups for migration and invasion assays (Transwell assays), with Dunnett’s or Sidak’s post-hoc tests performed depending on the experimental design. A p-value less than 0.05 was considered statistically significant (*p < 0.05, **p < 0.01, ***p < 0.001 and ****p < 0.0001).

## 3 Results

### 3.1 Cellular sensitivity of cannabinoids and their synergistic effects on ovarian cancer cells

**FIGURE 1:**
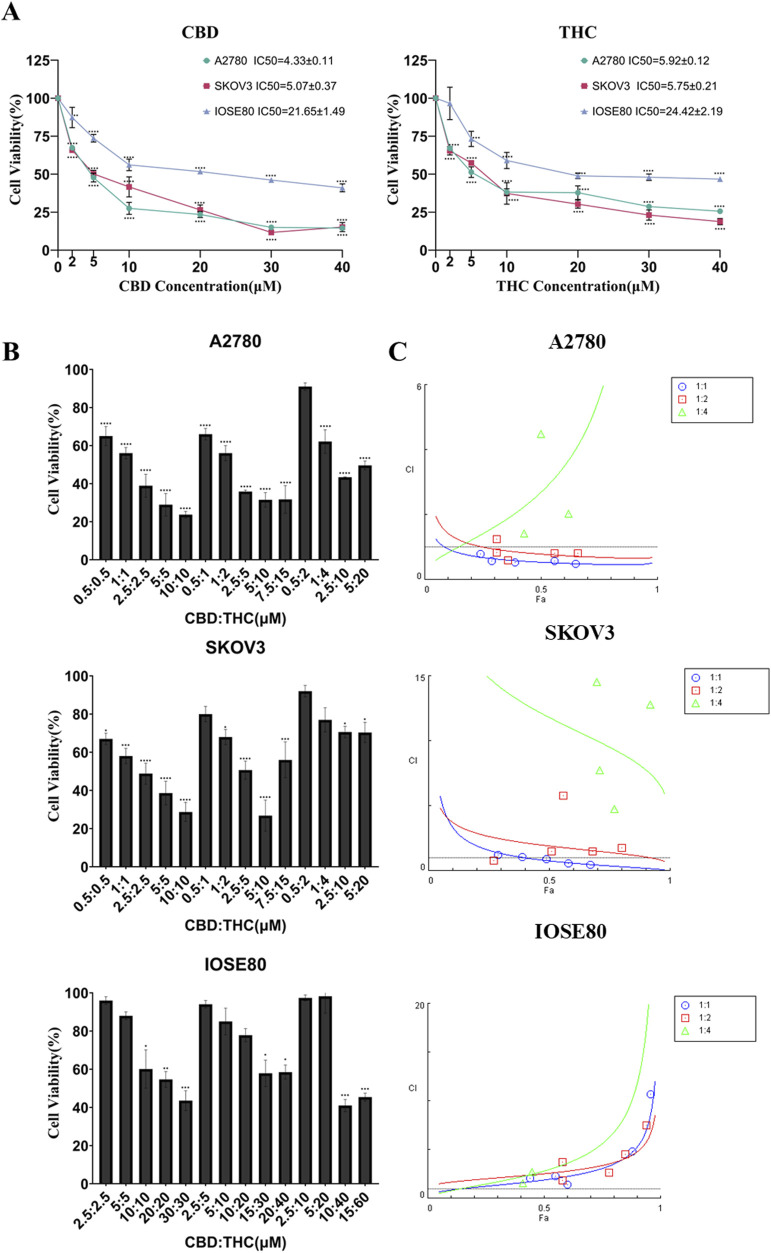
Anti-proliferative effects of CBD, THC, and their combinations on ovarian cancer cell lines and normal ovarian epithelial cells. **(A)** Dose-response curves of CBD (left) and THC (right) in A2780, SKOV3, and IOSE80 cell lines after 48-h treatment. Data are presented as mean ± SD (n = 3), statistical significance was analyzed using one-way ANOVA followed by Tukey’s post hoc test ****p < 0.0001 vs. control. **(B)** Combinatory effect of different CBD:THC combinations on cell viability in A2780, SKOV3, and IOSE80 cells. Data are presented as mean ± SD (n = 3), differences among groups were analyzed using one-way ANOVA followed by Dunnett’s post hoc test, *p < 0.05, **p < 0.01, ***p < 0.001 and ****p < 0.0001 vs. control. **(C)** Combination Index (CI) Combination index analysis using CompuSyn software for 1:1, 1:2, and 1:4 ratios of CBD: THC in A2780, SKOV3, and IOSE80 cells. CI < 1 indicates synergism, CI = 1 additive effect, and CI > 1 antagonism.

The viability of ovarian cancer cell lines (SKOV3 and A2780) and a non-tumorigenic ovarian cell line (IOSE80) were assessed after treatment with different concentrations of CBD and THC for 24, 48 and 72 h. Both CBD and THC inhibited the viability of cancer cells including A2780 and SKOV3 in a dose-dependent inhibitory manner. The remarkable cytotoxicity was noticed in SKOV3 and A2780, showing the strong inhibition, with the 48 h IC_50_ values 4.33 ± 0.11 µM and 5.07 ± 0.37 µM respectively, indicating the selective toxicity potency to cancer cells while compared to the non-cancer IOSE cells with 48 h IC_50_ value of 21.65 ± 1.49 µM (Figure 1A). The similar trend was found in THC treated cells in which THC considerably suppressed cell growth of cancer cells with notable 48 h IC_50_ values of 5.92 ± 0.12 µM, 5.75 ± 0.21 µM for A2780 and SKOV3, and 24.42 ± 2.19 µM in IOSE cells, highlighting the fact that cannabinoids are potent cytotoxic agents to cancer cells than to non-tumorigenic IOSE80 cells and supporting their potential safety credentials (Figure 1A).

We then evaluated the potential combinatorial effects of CBD and THC at different ratios on A2780, SKOV3, and IOSE cells (Figure 1B). The results showed that the synergistic treatment more pronouncedly suppressed the viability of cancer cells than the individual treatments, remarkably at lower ratio level (0.5:0.5, 2.5:2.5 µM), whereas a higher proliferation was observed in IOSE under the same treatments, validating their safety feature by triggering the minimal toxicity to non-cancer cells (Figure 1B).

### 3.2 Evaluation of CBD and THC drug interaction using Chou-Talalay method

To assess the nature of interaction between CBD and THC, we applied combination index (CI) parameter using the Chou-Talalay method (Chou and Talalay, 1984; Chou, 2018) at various fraction affected (Fa) including at 20%, 50%, and 80% cell death (Figure 1C; Table 1). In A2780 cells, the 1:1 CBD: THC ratio showed CI values of 0.7, 0.5, and 0.5 at 20%, 50%, and 80% effect levels in A2780 cells, respectively, demonstrating a notable synergy (Figure 1C; Table 1). In the treatment of 1:2, the result indicated an additive effect with Fa 20% (CI = 1.0) and synergistic effects with higher Fa values of 0.8 at Fa 50%, 0.7 at Fa 80%, indicating a concentration-dependent synergistic effect of CBD: THC treatment in A2780 cells (Figure 1C; Table 1). However, we observed an antagonistic effect in the 1:4 ratio at all Fa values, with CI increasing substantially with higher Fa values of1.2 at 20%, 2.7 at 50%, and 6.9 at 80%, respectively (Figure 1C; Table 1).

**TABLE 1:** Combination index (CI) parameters were determined by the chou-talalay method at multiple fractions affected (Fa), specifically at 20%, 50%, and 80% cell death.

Ratio (CBD: THC)/Cell lines	CI at fraction affected (%)		
	20	50	80
A2780			
1:1	0.7	0.5	0.5
1:2	1.0	0.8	0.7
1:4	1.2	2.7	6.9
SKOV3			
1:1	1.9	0.8	0.3
1:2	2.9	1.9	1.3
1:4	15.8	11.8	9.0
IOSE80			
1:1	1.1	1.9	3.5
1:2	1.9	2.5	3.7
1:4	1.2	2.8	6.8

The 1:1 CBD: THC treatment in SKOV3 cells exhibited an antagonistic effect at lower Fa value with CI = 1.9 at Fa 20%, switching to synergistic effects at higher Fa levels with 0.8 at Fa 50%, 0.3 at Fa 80% (Figure 1C; Table 1). This finding suggests the possibility of a dose-dependent interaction between CBD and THC, potentially involving differential engagement of molecular mechanism. We noticed an antagonistic effect (CI > 1) in the higher ratio of 1:2 at all Fa values with 2.9, 1.9, and 1.3 at 20%, 50%, and 80%, respectively, whereas the 1:4 ratio exhibited predominant antagonism (CI > 1) across all Fa values, with high CI values of15.8 at Fa 20%, 11.8 at Fa 50%, 9.0 at Fa 80% (Figure 1C; Table 1). In non-tumor IOSE80 cells, all CBD: THC ratios including 1:1, 1:2, and 1:4 showed additive to antagonistic effects with CI values greater than 1 across all Fa levels, demonstrating predominantly antagonistic interactions. For the 1:1 ratio, CI values were 1.1 at Fa 20%, 1.9 at Fa 50%, and 3.5 at Fa 80%, while CI values of 1.9 at Fa 20%, 2.5 at Fa 50%, and 3.7 at Fa 80% were observed at 1:2 ratio treatment (Figure 1C; Table 1). We found a similar trend in the 1:4 ratio which yielded CI values of 1.2, 2.8, and 6.8 at Fa 20%, 50%, and 80%, respectively, favoring a potential antagonistic interaction of CBD and THC and increased tolerance in non-tumor IOSE cells (Figure 1C; Table 1).

### 3.3 The cannabinoid combination suppressed colony formation and migration of ovarian cancer cells

**FIGURE 2:**
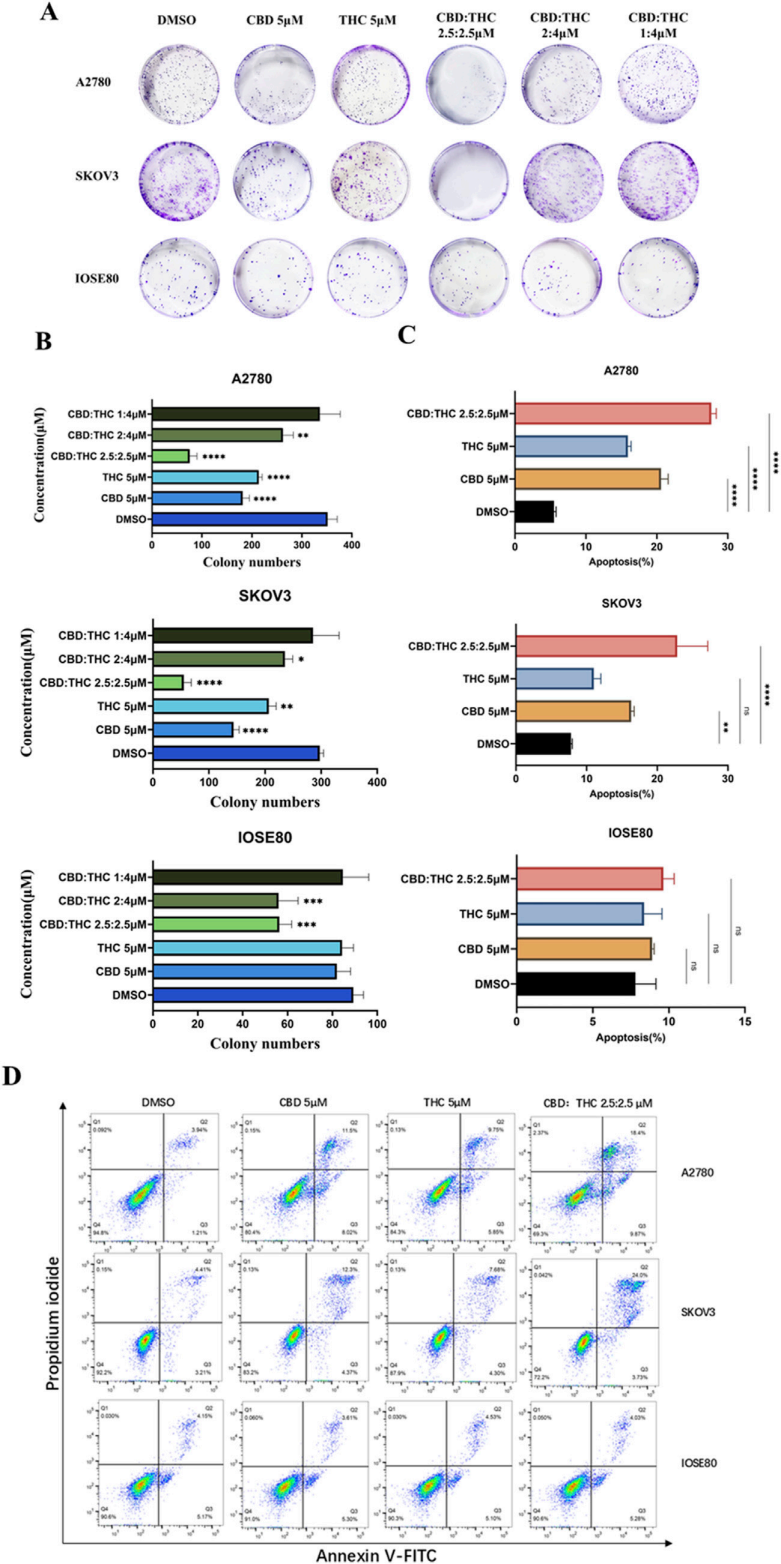
**(A)** Clonogenic assay of A2780, SKOV3, and IOSE cells treated with CBD, THC, and combination. Representative images of colony formation assay in A2780, SKOV3, and IOSE80 cells after treatment with DMSO, CBD (5 µM), THC (5 µM), and combinations of CBD: THC at different ratios. Cells were incubated and colonies stained with crystal violet were counted. **(B)** Quantification of colony numbers using ImageJ. The data are presented as the mean ± SD from at least three experiments, differences among groups were analyzed using one-way ANOVA followed by Dunnett’s post hoc test. *P*-value of <0.001 is represented as *** and of <0.0001 as ****. **(C)** The data provides the percentage of viable, early apoptotic and late apoptotic cells. Data are presented as the means ± SD of triplicate experiments, differences among groups were analyzed using one-way ANOVA followed by Dunnett’s post hoc test. *p < 0.05, **p < 0.01, ***p < 0.001 and ****p < 0.0001 vs. control. **(D)** Apoptosis assay using flow cytometry after staining with annexin V-FITC/propidium iodide.

To evaluate the long-term inhibitory effects of CBD, THC, and their combination on the proliferation of ovarian cancer cells, we conducted the colony formation assay. The clonogenic or colony formation assay assesses a cell’s ability to proliferate indefinitely, preserving its reproductive ability to form a large colony or clone. This cell is referred to as clonogenic (Munshi et al., 2005). Analysis of colony numbers resulted in a greatly reduced number and size of colonies after exposure with CBD and THC in ovarian cancer cells (A2780 and SKOV3) compared to the DMSO control cells, demonstrating a marked inhibitory effect of individual cannabinoid on colony formation (Figures 2A,B). In contrast, non-tumor IOSE cells exhibited a relatively consistent in colony numbers by demonstrating unremarkable change in the number and size when treated with CBD, THC, and combination, maintaining a higher colony-forming ability (Figures 2A,B). Interestingly, the treatment with CBD: THC 2.5:2.5 µM revealed a significantly decreased colony formation in both ovarian cancer cell lines (A2780 and SKOV3) and attenuated the proliferation of cancer cells by inhibiting clonogenic potential, highlighting a powerful synergistic inhibitory effect, particularly in A2780 cell line showing the most prominent inhibition with p<0.0001 (Figures 2A,B). For IOSE cells, we observed fewer reductions of colony numbers in combination treatments (CBD: THC) than the cancer cells, and a minimal impact was observed when treated with CBD or THC alone at 5 μM, indicating a lower susceptibility to the treatments compared to the cancer cell lines (Figures 2A,B).

### 3.4 Apoptosis induction of CBD: THC combination on ovarian cancer cells

To investigate whether the observed inhibition of the compounds (CBD, THC), and their combination on ovarian cancer cells was associated with apoptosis induced cell cycle arrest, we conducted flow cytometry analysis after staining with Annexin V-FITC and Propidium Iodide (PI) (Figures 2C,D). Annexin V identifies cells in both early and late stages of apoptosis, unlike PI which stains the cells only in late apoptosis or necrosis. Early apoptotic cells will be positively stained for Annexin V and negatively stained for PI (lower right quadrant); whereas those in late apoptosis cells will exhibit positive staining for both Annexin V and PI (upper right quadrant).

A moderate increase in the number of apoptotic cells was observed in A2780 cells, after treatment with 5 μM of CBD or THC alone and their combination (CBD: THC 2.5:2.5 μM) for 48 h compared to the DMSO control. However, the combination treatment showed significantly induced apoptosis percentage reaching approximately 25% which was notably higher than either CBD or THC alone, signifying its pro-apoptotic effects on cancer cells (Figures 2C,D). Interestingly, a similar trend was observed in SKOV3 cells, where the CBD: THC 2.5:2.5 μM combination exhibited the most pro-apoptotic effect, with approximately 28%, although single treatment with CBD and THC also showed considerably increased apoptosis impact on the cancer cells, validating the potential therapeutic efficacy of combined treatment (Figures 2C,D). In contrast, either of CBD or THC treatment did not induce apoptosis with the same concentrations in non-tumor IOSE cells, while a slight increase in apoptosis percentage was noted with the combination treatment, suggesting the selectivity of enhanced apoptosis on cancer cells and lower susceptibility of non-tumor cells to cannabinoid-induced apoptosis (Figures 2C,D).

### 3.5 CBD: THC combination treatment induced G0/G1 cell cycle arrest in ovarian cancer cells

**FIGURE 3:**
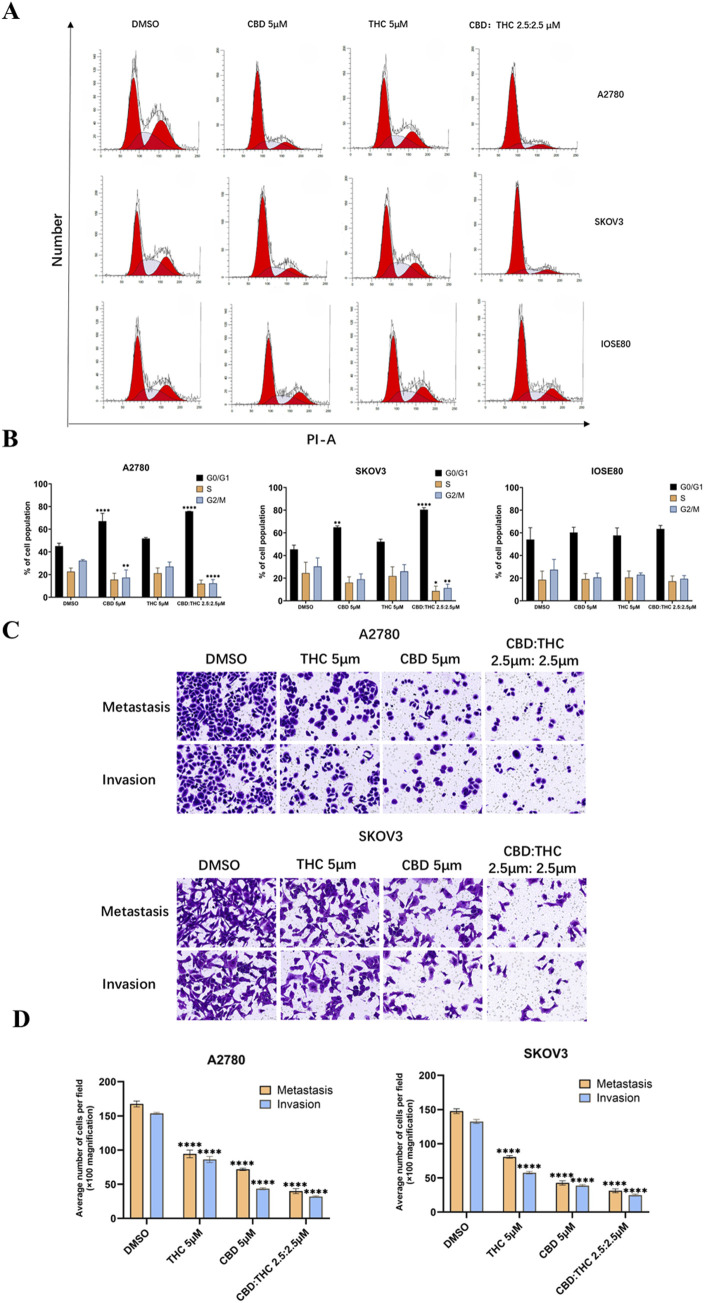
Effects of CBD, THC, and their combination on cell cycle distribution, and metastasis behavior in ovarian cancer cells. **(A)** Cell cycle analysis using propidium iodide (PI) staining and flow cytometry following 48-h treatment with DMSO (control), CBD (5 µM), THC (5 µM), or a combination of CBD: THC (2.5:2.5 µM). **(B)** Percentage of cells in G0/G1, S, and G2/M phases. Data are presented as the mean ± SD of triplicate experiments. Statistical analysis was performed by two-way ANOVA followed by Sidak’s post hoc test (*p < 0.05). **(C)** Illustration of Transwell cell migration and invasion assay. **(D)** ImageJ quantified the relative number of migrating and invading cells. Data are presented as mean ± SD (*n* = 3). Statistical analysis was performed by two-way ANOVA followed by Dunnett’s post hoc test. *P<0.05, compared with the DMSO control group.

The cell cycle is a crucial regulator of cell proliferation and growth after damaging of DNA; hence the cell cycle can protect the cell from DNA damage, and it is also the important survival mechanism of tumor cells. Thus, one of the target anticancer therapies is to inhibit the cell cycle which may induce apoptosis, giving rise to cancer cell death (Schwartz and Shah, 2005). Therefore, we investigated the effects of CBD: THC combination on the cell cycle progression of ovarian cancer cells, along with individual treatment using flow cytometric analysis after PI staining in A2780, SKOV3, and IOSE cells. In both ovarian cancer cell lines (A2780 and SKOV3), treatment with either CBD (5 µM) or THC (5 µM) alone resulted in significant accumulation of cells in the G0/G1 phase, while attenuating the accumulation of cells in the S and G2/M phases (Figures 3A,B). Notably, combined CBD: THC treatment (2.5:2.5 µM) further increased the number of cells in the G0/G1 phase compared to treatment with either CBD or THC alone, demonstrating that CBD and THC exhibit synergistic effects on cell cycle progression in ovarian cancer cells (Figures 3A,B).

In A2780 cells, treatment with either CBD or THC (5 μM) alone increased the number of cells in the G0/G1 phase and decreased the number in the S and G2/M phases. Compared to the control group, the control group showed a more typical cell cycle distribution, with approximately 40% of cells in the G1/G0 phase and approximately 60% in the S and G2/M phases (Figures 3A,B). Combined treatment with CBD: THC also showed a significant increase in the number of cells in the G0/G1 phase and a decrease in the number of cells in the S and G2/M phases (Figures 3A,B), indicating that CBD, THC, and their combination effectively induce cell cycle arrest in G1/G0 in both ovarian cancer cell lines (Figures 3A,B). On the other hand, treatment with CBD, THC, or their combination did not significantly alter the cell cycle distribution in the G0/G1, S, or G2/M phases in non-tumor IOSE cells, suggesting that cannabinoids selectively target the cancer cell cycle (Figures 3A,B).

### 3.6 The combination of CBD and THC resulted in the suppression of ovarian cancer cell migration and invasion

We assessed the effect of cannabinoids on cell migration and invasion using Transwell cell migration and Matrigel invasion assays and the results were quantified using ImageJ software to count the relative number of cells. In A2780 cells, after induction with CBD (5 μM) and THC (5 μM) for 48 h, the migration and invasion abilities of the cancer cells were significantly decreased compared to DMSO treated control cells (Figures 3C,D). Notably, the combination treatment of CBD (2.5 μM) and THC (2.5 μM) showed a more profound impact than individual treatments by strongly suppressing the migration and invasion of the cancer cells (Figures 3C,D). A similar trend was noted in SKOV3 cells, where treatment with CBD or THC alone (5 µM) demonstrated considerably lowered migration and invasion, with the combination providing the most dominant inhibitory effect (Figures 3C,D), validating the potent effectiveness of CBD: THC combination on the impairment of metastatic behavior in cancer cells.

### 3.7 The CBD: THC combination induced mitochondrial membrane potential depolarization

**FIGURE 4:**
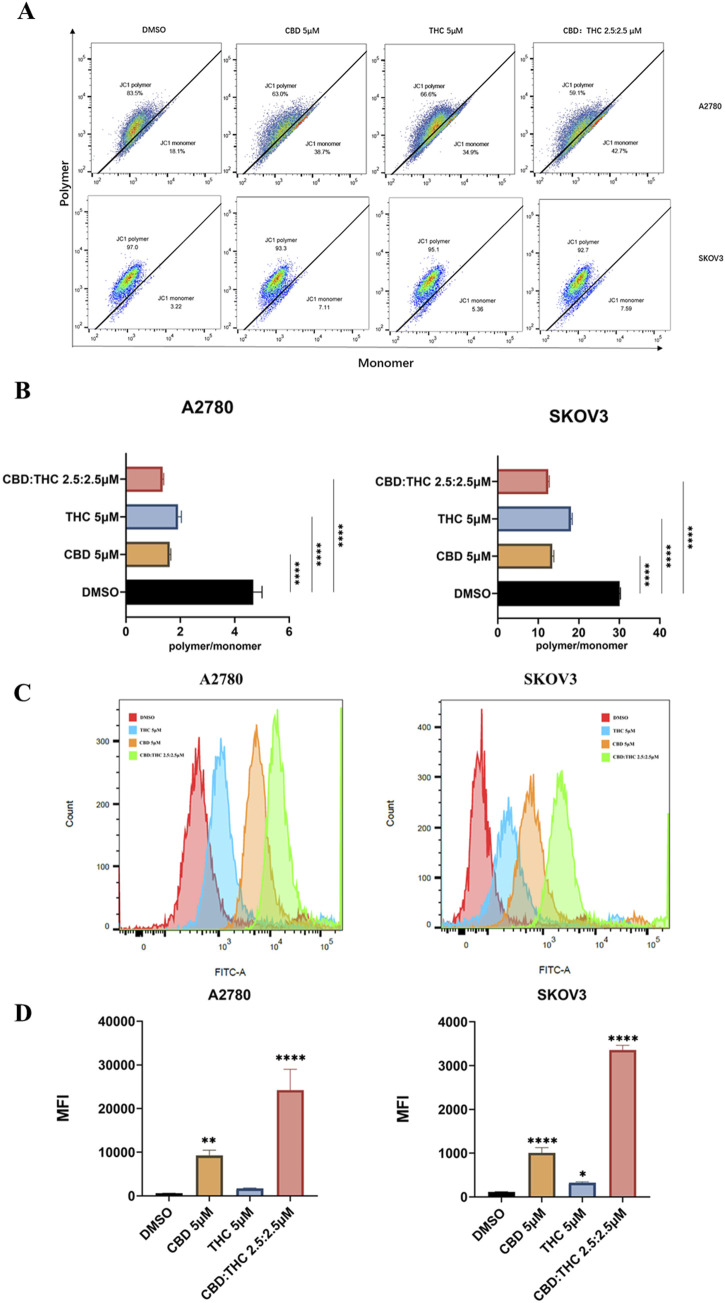
Assessment of CBD, THC, and their combination on mitochondrial depolarization using JC-1 and determination of cellular reactive oxygen species (ROS). **(A)** Representative flow cytometry plots for mitochondrial membrane potential. Ovarian cancer cells were treated with DMSO (control), CBD (5 μM), THC (5 μM), and CBD: THC (2.5:2.5 μM) for 48 h. MMP was assessed using JC-1 staining. The x-axis represents JC-1 monomer (green fluorescence), and the y-axis represents JC-1 polymer (red fluorescence). **(B)** The ratio of JC-1 polymer to monomer fluorescence intensity was quantified after 48 h of treatment. A lower polymer/monomer ratio indicates mitochondrial depolarization. **(C)** Representative histograms demonstrating the ROS level of ovarian cancer cells after treatment with cannabinoids using flow cytometry. **(D)** Quantitative analysis of ROS levels in **(C)**. Data are presented as mean ± SD of three independent experiments. Statistical significance was determined by one-way ANOVA followed by Dunnett’s post-hoc tests (****p<0.0001 compared to DMSO control).

We evaluated the impact of cannabinoids and their combination on mitochondrial membrane potential (ΔΨm) using JC-1 staining and flow cytometry in A2780 and SKOV3 ovarian cancer cells. The changes in the red/green fluorescence intensity ratio (polymer/monomer ratio) indicate mitochondrial depolarization, whereas red-fluorescent aggregates (JC-1 polymer) in healthy mitochondria and green-fluorescent monomers in depolarized mitochondria. DMSO-treated control cells showed a high JC-1 polymer-to-monomer ratio, indicating the intact mitochondrial potential while cannabinoid treated cells (either CBD or THC) demonstrated a noticeable shift in both cells (A2780 and SKOV3) from JC-1 polymer (red fluorescence) to JC-1 monomer (green fluorescence), resulting in a moderate loss of ΔΨm by demonstrating a significant decrease in the polymer/monomer ratio (Figures 4A,B). Notably, the flow cytometry plots of the CBD: THC combination (2.5:2.5 µM) produced the most remarkable mitochondrial depolarization in both ovarian cancer cells compared to individual treatment, corresponding to a higher proportion of JC-1 monomers by disrupting mitochondrial integrity (Figures 4A,B).

### 3.8 The CBD: THC combination induces mitochondrial ROS (mROS)

Mitochondria is a major source of intracellular ROS in most cell types (Murphy, 2009), so we used MitoSOX Red (MitoSOX) to detect mitochondrial ROS. To verify whether disruption of mitochondrial function often results in reactive oxygen species (ROS) production, we detected ROS generation in ovarian cancer cells using flow cytometry after incubation with 5 µM of CBD or THC, and 2.5:2.5 µM of CBD: THC. We observed a clear rightward shift in the fluorescence peaks in cannabinoids treated cells compared to the DMSO control, especially in the CBD: THC combination treatment. Cannabinoids were found to increase MitoSOX Red fluorescence signals in a treatment-dependent manner (Figures 4C,D). Interestingly, the combination treatment of CBD: THC represented the good synergistic impact of cannabinoids in promoting oxidative stress, elevating strong ROS production and significant induction of oxidative stress in A2780 and SKOV3 cells with over a 10-fold and 3-fold increase in mean fluorescence intensity, respectively, compared to control and CBD, or THC alone, a (Figures 4C,D).

### 3.9 The CBD: THC combination reactivates tumor suppressive signaling in ovarian cancer cells

**FIGURE 5:**
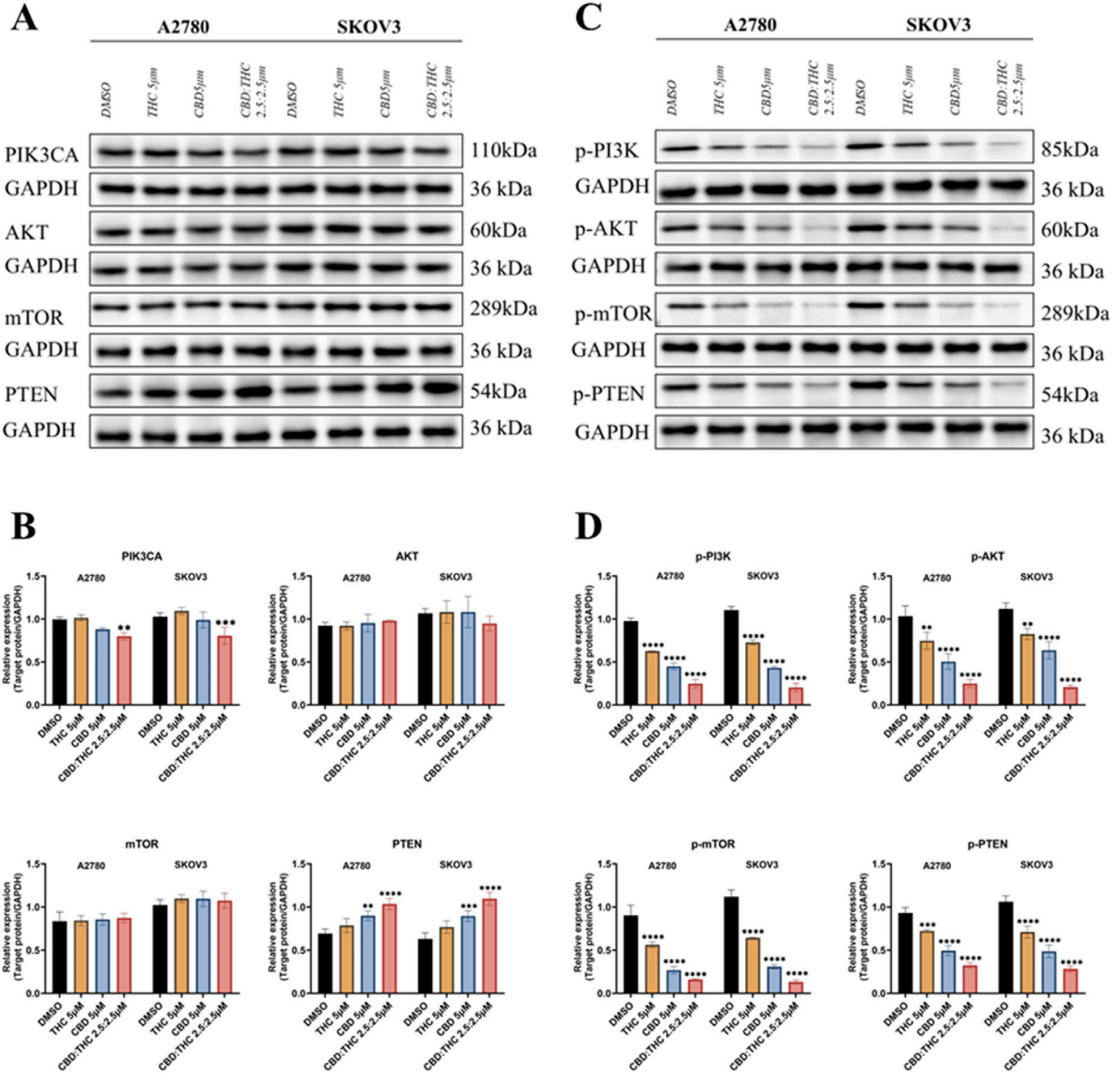
Molecular mechanisms underlying cannabinoids inhibition of ovarian cancer cell growth. **(A)** The expression levels of total PIK3CA, AKT, mTOR, and PTEN in A2780 and SKOV3 cells treated with DMSO (control), CBD (5 μM), THC (5 μM), and CBD: THC (2.5:2.5 μM). GAPDH was used as a loading control. **(B)** Densitometric analysis of total PIK3CA, AKT, mTOR, and PTEN protein levels, normalized to GAPDH, for A2780 and SKOV3 cells. **(C)** The phosphorylated protein expression levels of PIK3CA (p-PI3K), AKT (p-Akt), mTOR (p-mTOR), and PTEN (p-PTEN) in A2780 and SKOV3 cells treated with DMSO (control), CBD (5 μM), THC (5 μM), and CBD: THC (2.5:2.5 μM). GAPDH was used as a loading control. **(D)** Densitometric analysis of phosphorylated p-PI3K, p-AKT, p-mTOR, and p-PTEN protein levels, normalized to GAPDH, for A2780 and SKOV3 cells. Data are presented as mean ± SD of three independent experiments. Statistical significance was determined by two-way ANOVA followed by Sidak’s post-hoc tests. Asterisks indicate statistical significance.

To explore the molecular mechanisms by which cannabinoids inhibit ovarian cancer cell growth, we focused on the PI3K/Akt pathway that has been reported as the major signaling cascades implicated in tumor development and triggered by receptor tyrosine kinases (RTKs) and PI3K (Jiang and Ji, 2019; Glaviano et al., 2023) and is frequently hyperactivated in various cancers, including ovarian cancer, promoting cell proliferation, survival, and growth (Ghoneum et al., 2020). We analyzed the involvement of PI3K, Akt, mTOR in the PI3K/AKT/mTOR signaling pathway using Western blot. Figure 5 showed that the expression levels of total PIK3CA, AKT, mTOR, and PTEN in A2780 and SKOV3 cells treated with DMSO (control), CBD (5 μM), THC (5 μM), and CBD: THC (2.5:2.5 μM). GAPDH was used as a loading control. We found that the expression levels of Akt and mTOR did not show any significant changes after treatment with CBD, THC, or combination of CBD: THC in both cancer cell lines, whereas the combination treatment of CBD: THC notably suppressed PIK3CA expression in both cell lines compared to DMSO control and individual treatment (Figures 5A,B). On the other hand, PTEN, a well-known tumor suppressor, was upregulated in the cells treated with CBD, or CBD: THC combination, with the noticeable increase observed in SKOV3 cells, indicating reactivation of this tumor suppressor upon treatment (Figures 5A,B).

### 3.10 Blockade of PI3K/AKT/mTOR signaling by CBD: THC combination in ovarian cancer cells

The PI3K/AKT/mTOR pathway is primarily regulated by phosphorylation (Sarbassov et al., 2005; Glaviano et al., 2023), we further determined the effects of CBD, THC, and their combination on the phosphorylation status of key players of pathway activation (Figures 5C,D). The Figures 5C,D represents the relative phosphorylated protein expression after normalizing to GAPDH, the result showed that all the treatments with CBD, THC, and the CBD: THC 2.5:2.5 μM combination considerably suppressed the phosphorylation of PI3K (p-PI3K), AKT (p-AKT), and mTOR (p-mTOR) in both cell lines. Remarkably, the combination treatment consistently demonstrated the most striking inhibitory effect on the phosphorylation of pro-survival key proteins (p-PI3K, p-Akt, p-mTOR). Interestingly, cannabinoid treatment upregulated PTEN levels but significantly decreased its phosphorylation status, particularly in the combined treatment (Figures 5C,D), confirming enhanced activation of this negative regulator of the PI3K pathway. The observed increase in total PTEN expression together with a reduction in its phosphorylated form indicates that cannabinoids may enhance PTEN activity, contributing to suppression of the PI3K/AKT/mTOR signaling cascade.

## 4 Discussion

Our study demonstrated a comprehensive exploration into the anti-cancer effects of cannabidiol (CBD), delta-9-tetrahydrocannabinol (THC), and their combination (CBD: THC) on ovarian cancer cells (A2780 and SKOV3) and a non-tumor ovarian cell line (IOSE80). We found that individual treatment of CBD and THC exhibit selective cytotoxicity towards ovarian cancer cells, with significantly dose-dependent lower IC_50_ values compared to the non-tumor IOSE80 cells, supporting a favorable therapeutic index where cannabinoids can effectively target cancer cells (Hanker et al., 2012; Mihanfar et al., 2019). This selective toxicity finding is consistent with other published research, which has also reported the ability of cannabinoids to induce apoptosis and attenuate proliferation in various cancer cell lines while showing minimal effects on non-malignant cells (Galve-Roperh et al., 2000; Śledziński et al., 2023). Importantly, we investigated the potential for synergistic interactions between CBD and THC. Using the Chou-Talalay method, we observed a marked synergistic effect at equimolar low concentration with the 1:1 CBD: THC ratio in A2780 cells, particularly at higher fractions of cell death (Fa). This synergy was not the same across the cell lines and ratios; in SKOV3 cells, the 1:1 ratio demonstrated synergy only at higher Fa values, and other ratios often produced antagonistic effects. The most captivating finding of our study was the precise concentration- and ratio-dependent nature of the synergistic interaction, which underscores the complexity of cannabinoid-based therapies, highlighting the importance of precise dosing and ratio optimization for combination of CBD: THC-based therapy, a finding that should be carefully considered in future research (Chou and Talalay, 1984; Chou, 2010).

In particular, the remarkable shift from antagonism to synergy observed in the combination index under the combined treatment of CBD: THC in SKOV3 cells may suggests that the possibility of dose-dependent interactions (Schoeman et al., 2020; Tomko et al., 2020). At lower inhibition scores, CBD and THC may partially compete with overlapping receptors and signaling pathways, such as CB1/CB2 receptor-mediated modulation, leading to reduced combined efficacy (O’Reilly et al., 2023; Faiz et al., 2024) As therapeutic efficacy increases, higher effective concentrations may overcome initial competitive interactions and simultaneously active complementary anti-survival mechanisms, including enhanced mitochondrial ROS production and inhibition of the PI3K/AKT/mTOR signaling cascade, resulting in synergistic cytotoxicity (Faiz et al., 2024; Ye et al., 2024). The predominantly antagonistic interactions found in non-tumor IOSE cells further validate the safety advantage of the CBD: THC combination, as it suggests a reduced risk of toxicity to healthy cells (Blagosklonny, 2023). These observations highlight the complexity of the CBD and THC interactions and suggest the need for further mechanistic studies to dissect the molecular basis of this dynamic response.

The clonogenic assay further verified the cytotoxic potential of both individual treatment with CBD and THC to alleviate the long-term survival of cancer cells. More importantly, the CBD: THC treatment combination exhibited a profound inhibition, particularly at equimolar concentrations (CBD: THC 2.5:2.5 µM), indicating a favorable synergistic interaction. This finding agrees with previously published work in which cell-viability assays and combination-index analyses demonstrated synergistic or additive anti-cancer effects of cannabinoids, thereby reinforcing the rationale for cannabinoid therapy in cancer (Scott et al., 2014; Mazuz et al., 2020). In addition to their anti-proliferative effects, the metastatic cascade, which consists of cell migration and invasion, is a major challenge in ovarian cancer treatment and is responsible for patient mortality (Hanker et al., 2012; Marth et al., 2017). Our Transwell assays clearly demonstrated that CBD and THC significantly suppressed both the migratory and invasive potential of A2780 and SKOV3 cells. The ability of cancer cells to migrate through extracellular matrix and invade surrounding tissues is a prerequisite for metastasis (Hanahan and Weinberg, 2011). The observed inhibition in both migration and invasion may suggest that these cannabinoids can hinder key steps in the metastatic process. The mechanisms underlying this inhibition could involve modulation of focal adhesion kinases, matrix metalloproteinases (MMPs), or epithelial-mesenchymal transition (EMT) pathways, which are all crucial for cell motility and invasion (Lu and Kang, 2019; Akhlaghipour and Moghbeli, 2024). Future studies should delve into these molecular targets to fully elucidate the anti-metastatic mechanisms. This nature of cannabinoids (CBD and THC) whether it is alone or in combination with each other attenuated the long-term proliferative and metastatic potential of ovarian cancer cells. This is a noteworthy finding of our study, as the power to suppress colony formation and cell migration is directly linked to a compound’s anti-cancer ability and its potential to prevent tumor recurrence and metastasis (Li et al., 2015; Hou et al., 2021).

The majority of non-viable cells showed signs of early or late apoptosis, with only small percentages of cells being necrotic. Necrosis refers to a type of cell death that triggers an inflammatory response and, therefore, can harm surrounding tissue, whereas apoptosis is a highly regulated process that eliminates a cell without damage to its neighboring cells. Therefore, many anticancer therapies aim to induce apoptosis as a key mechanism to eliminate cancer cells. However, in cancer cells, the apoptotic machinery often becomes dysfunctional. Alterations in the extrinsic and intrinsic apoptosis pathways, as well as disruptions in the balance between pro-apoptotic and anti-apoptotic molecules, enable cancer cells to survive, leading to tumor growth and progression (Pistritto et al., 2016). Therefore, we conducted flow cytometry to evaluate apoptotic cell populations following compound exposure.

Flow cytometric analysis provided the critical insights into the mechanism underlying the observed anti-proliferative action of cannabinoids treated cells. The significant induction of apoptosis by CBD, THC, and especially their combination, in both A2780 and SKOV3 ovarian cancer cell lines, revealed that programmed cell death is a major contributor to the decreased cell viability and colony formation. The CBD: THC combination demonstrated a more potent pro-apoptotic effect, consistently triggering a higher percentage of cell death than individual CBD or THC treatments. This synergistic outcome indicated that the cannabinoids likely modulate separate yet cooperative apoptotic cascades, enhancing their combined therapeutic impact, and offering a desirable characteristic for an an-cancer therapy (Qin et al., 2018; Chaudhary et al., 2025). By demonstrating minimal apoptosis in non-tumor IOSE cells while inducing significant effects in cancer cells, the comparative analysis further validated the selective toxicity of the CBD and THC combination. This differential apoptotic response is a critical consideration in therapy development that protect healthy cells from harm while specifically attack cancer cells, a key component of a favorable therapeutic index (Fischer and Schulze-Osthoff, 2005; Blagosklonny, 2023). The mechanisms underlying this selectivity could be attributed to differences in the expression of cannabinoid receptor, distinct cellular uptake mechanisms, unique metabolic pathways, or the activation of pro-survival and pro-apoptotic signaling cascades in normal and cancerous cells (Cianchi et al., 2008; Soderstrom et al., 2017; Ramer et al., 2019; Kovalchuk and Kovalchuk, 2020; Pagano et al., 2021; Song et al., 2023). For instance, cancer cells often showed dysregulated signaling pathways that make them more susceptible to apoptotic triggers, which cannabinoids might exploit (Shrivastava et al., 2011; Armstrong et al., 2015; Velasco et al., 2016).

Cell cycle analysis revealed a potent G1/G0 phase arrest in A2780 and SKOV3 ovarian cancer cells after treatment with CBD or THC. Noteworthily, the combination of CBD and THC, even at half their individual concentrations (2.5 μM each), effectively induced cell cycle arrest, highlighting a potential synergistic or additive interaction between CBD and THC to achieve powerful anti-cancer effects at lower cumulative doses. The increased cell population in the G0/G1 phase suggests that the treatments halted cell cycle progression before the initiation of DNA replication and mitosis, effectively suppressing the uncontrolled proliferation typical of cancer cells (Ye et al., 2003; Yam et al., 2022). The induction of cell cycle arrest is fundamental to the action of many anti-cancer agents (Visconti et al., 2016). This consistent anti-proliferative response in both the A2780 and SKOV3 cell lines strongly suggested that the underlying mechanism is not cell-line specific but rather an a fundamental property of the cannabinoids (Munson et al., 1975; Freimuth et al., 2010). In stark contrast to the effects on cancer cells, CBD, THC, and their combination did not significantly disrupt the cell cycle of non-tumor IOSE cells, which is a very promising result of our study, validating their selective toxicity towards cancer cells as a potential therapeutic agent. Possible explanations for this may include varying levels of cannabinoid receptor expression, divergent downstream signaling cascades, or altered metabolic characteristics between the normal and cancerous cells (Lakiotaki et al., 2015; Ibsen et al., 2017; Ramer et al., 2019; Vidlarova et al., 2022; Wnorowski et al., 2022).

Although our results demonstrated selective toxicity of cannabinoids toward ovarian cancer cells, the IC_50_ value of ∼24.42 µM in IOSE80 non-tumor cells indicates mild to moderate toxicity at higher concentrations, which may limit the therapeutic window. However, this concentration likely exceeds plasma levels typically achieved *in vivo* following clinically relevant cannabinoid dosing (Millar et al., 2018). Studies have reported similar selectivity profiles, for example, training CBD paralytics in non-tumor cell lines report IC_50_s in the range between 20–40 μM, while much lower concentrations are active in cancer cell models (Shrivastava et al., 2011; Ramer et al., 2019). These findings suggest a dose-dependent behavior of cannabinoids *in vitro* where cytotoxic effects on normal cells occur predominantly at suprapharmacological concentrations. However, *in vitro* data should be interpreted cautiously, since cellular uptake, protein binding, metabolism, and distribution differ substantially *in vivo* (Bell et al., 2018). Furthermore, we did not include an ADMET (Absorption, Distribution, Metabolism, Excretion, Toxicity) assessment. While in-depth ADMET profiling was beyond the scope of this mechanistic *in vitro* work, we acknowledge this omission as a limitation. Future investigations are warranted to perform *in silico* and *in vitro* ADMET modeling and pharmacokinetic simulations to evaluate the factors such as oral bioavailability, metabolic stability, and toxicity (Wang et al., 2015; Wu et al., 2020) of the CBD: THC combination. Such analyses will offer a more comprehensive understanding of translational feasibility, dosing safety, and therapeutic index of cannabinoid-based therapies (Hines et al., 2022).

Mitochondrial dysfunction and ROS accumulation are hallmark in inducing apoptosis and inhibiting cancer cell proliferation. A healthy MMP is essential for ATP production and cellular homeostasis. Its disruption leads to the release of pro-apoptotic factors (e.g., cytochrome c) from the mitochondria into the cytoplasm, ultimately activating caspases and initiating programmed cell death (Henry-Mowatt et al., 2004; Burke, 2017). Our findings demonstrated that the combination of CBD and THC contributed to a significant disruption in mitochondrial membrane potential in ovarian cancer cells compared to individual treatment with either CBD or THC, indicating that the combined treatment triggered mitochondrial stress as an early pro-apoptotic signal. In addition, we observed an enhanced ROS generation after treatment with cannabinoids, particularly with the CBD: THC combination. ROS are highly reactive molecules that, at elevated levels, can cause oxidative damage to cellular components, including DNA, proteins, and lipids, leading to cellular dysfunction and death. While low levels of ROS can act as signaling molecules, excessive ROS accumulation triggers oxidative stress, which can directly induce mitochondrial dysfunction and initiate apoptotic pathways (Murphy, 2009; Schieber and Chandel, 2014). The synergistic action of CBD and THC resulted in a marked increase in ROS production, likely due to direct ROS generation or the impairment of antioxidant defense systems, thereby pushing the cancer cells beyond their oxidative stress tolerance level (Schieber and Chandel, 2014; Burke, 2017; Bustamante-Barrientos et al., 2025).

The PI3K/AKT/mTOR signaling pathway is frequently hyperactivated in ovarian cancer, contributing to uncontrolled cell proliferation, survival, and resistance to therapy (Ghoneum et al., 2020; Rinne et al., 2021). We found that the combination of CBD and THC effectively inhibited this oncogenic signaling cascade in A2780 and SKOV3 ovarian cancer cell lines. Our Western blot results demonstrated that the total protein levels of Akt and mTOR remained largely unchanged after treatment with CBD: THC, whereas the phosphorylation status of these (p-Akt and p-mTOR), along with p-PI3K, were downregulated, indicating that combination treatment may disrupt the activation of pathway without altering the baseline protein levels. Notably, this inhibition was synergistic, with the combination producing a more substantial reduction than treatment with either CBD or THC alone.

More importantly, the combination treatment (CBD: THC) resulted in a significant modulation of PTEN and its phosphorylated form (p-PTEN) expression. Our results showed a decrease in phosphorylated PTEN levels accompanied by an increase in total PTEN protein levels, suggesting functional restoration of this tumor suppressor. PTEN primarily exerts its inhibitory effect on the PI3K/AKT pathway by dephosphorylating phosphatidylinositol (3,4,5)-trisphosphate (PIP3) to PIP2. Phosphorylation of PTEN at the C-terminus at Ser380, Thr382, and Thr383 induces a “closed” confirmation, enhancing stability but reducing catalytic activity: dephosphorylation of these residues, on the other hand, promotes an “open” conformation, facilitating it catalytically active and enhancing membrane binding (Song et al., 2012). Therefore, the observed decrease in PTEN phosphorylation in our study may reflect a conformational shift toward the catalytically active state, enabling it to more effectively antagonize PI3K/AKT signaling. This effect, combined with increased total protein, could enhance tumor suppressor signaling and promote apoptosis. Mechanistically, these effects may be driven by the regulation of upstream kinases and phosphatases. Casein kinase 2 (CK2) has been shown to phosphorylate the C-terminal cluster of PTEN, while phosphatases such as PP2A can dephosphorylate these residues, promoting its active conformation. Furthermore, cannabinoid-induced ROS generation and CB1/CB2 receptor activation may indirectly affect PTEN activity through post-translational modifications or upstream regulatory cascades (Lakiotaki et al., 2015; Song et al., 2023). In addition, differential expression or coupling efficiency of CB1 and CB2 receptors among ovarian cancer cell lines may contribute to the distinct responses observed between cancer cells (Velasco et al., 2016; Moreno et al., 2019). Previous studies have shown that variations in receptor density and downstream G-protein signaling can influence cannabinoid-induced modulation of the PI3K/AKT/mTOR pathway and apoptotic sensitivity (Velasco et al., 2016; Moreno et al., 2019). These mechanisms may collectively explain the observed increases in total PTEN protein and decreases in p-PTEN levels, as well as the cell line-specific responses observed between A2780 and SKOV3 cells (Lee et al., 2002; Song et al., 2012; Bai et al., 2015; Elias et al., 2015; Zheng et al., 2023). Restoration of PTEN function is often associated with improved therapeutic response and attenuated oncogenic signaling (Lin et al., 2021). Therefore, our findings suggest that the CBD: THC combination not only hinders pro-survival PI3K/AKT/m-TOR signaling but also reactivates intrinsic tumor suppressor pathways, offering a dual-targeted approach for the treatment of ovarian cancer. While the observed increase in total PTEN protein accompanied by decreased phosphorylation at the S380/Thr382/Thr383 cluster suggests potential modulation of PTEN activity, direct measurements of its lipid phosphatase function was not performed in this study. Further investigations employing PTEN activity assays or PIP3 quantification are warranted to confirm the functional consequences of these molecular changes. This mechanistic insight supports the potential of cannabinoid-based therapeutic strategies in precision oncology and warrants further investigation of PTEN regulation and activity in future studies.

It was worth noting that phosphorylation of the C-terminal S380/Thr382/Thr383 cluster promotes PTEN stability but suppresses its catalytic activity by maintaining a “closed” conformation. Therefore, the concurrent increase in total PTEN and decrease in phosphorylation observed in this study may appear paradoxical, as phosphorylation is known to stabilize PTEN while hindering its activity. This pattern suggests that additional stabilization mechanisms, such as reduced ubiquitin-mediated degradation and transcriptional upregulation, may compensate for the loss of phosphorylation-dependent stability. Consequently, the overall effect may favor a more active, dephosphorylated PTEN conformation, enhancing its tumor suppressor function although decreased phosphorylation. Future studies should assess PTEN half-life and direct phosphatase activity to clarify this regulatory balance. Taken together, these findings highlight the complex, multilayered regulation of PTEN by cannabinoids and support further investigation of cannabinoid-mediated modulation of PI3K/AKT/mTOR signaling in ovarian cancer.

## 5 Conclusion

Our study elucidated the multi-faceted anti-cancer properties of cannabidiol (CBD) and delta-9-tetrahydrocannabinol (THC), particularly in their combination treatment, by demonstrating potent and selective anti-cancer activities against ovarian cancer cells without harming non-tumor IOSE cells, establishing a favorable therapeutic index. The combination treatment of CBD and THC exhibited concentration- and ratio-dependent synergy, inhibiting proliferation, and hindering metastatic potential through impaired migration and invasion while inducing apoptosis and attributing to mitochondrial membrane depolarization. Mechanistically, we revealed that CBD and THC, particularly the CBD: THC combination effectively suppresses the PI3K/AKT/mTOR signaling axis by downregulating the phosphorylation of p-PI3K, p-Akt, and p-mTOR, whereas restoring the function of the tumor suppressor PTEN. This dual modulation of oncogenic and tumor-suppressive pathways endorses the therapeutic potential of CBD: THC treatment as a targeted anti-cancer strategy. Our findings warrant further in functional phosphatase activity to confirm the reactivation of PTEN lipid phosphatase enzyme, and vivo validation and clinical exploration to optimize cannabinoid-based regimens for ovarian cancer treatment, especially considering the precise concentration- and ration-dependent nature of their interactions.

## Data Availability

The original contributions presented in the study are included in the article/Supplementary Material, further inquiries can be directed to the corresponding author.
